# Current Practice of Stress Ulcer Prophylaxis in Surgical Departments in Mecklenburg Western Pomerania, Germany

**DOI:** 10.3390/healthcare9111490

**Published:** 2021-11-02

**Authors:** Julia Rauch, Marco Franze, Maciej Patrzyk, Claus-Dieter Heidecke, Tobias Schulze

**Affiliations:** 1 Department of General Surgery, Visceral, Thoracic and Vascular Surgery, Universitätsmedizin Greifswald, 17475 Greifswald, Germany; julia.rauch@stud.uni-greifswald.de (J.R.); patrzyk@uni-greifswald.de (M.P.); heidecke@uni-greifswald.de (C.-D.H.); 2 Institute of Community Medicine, Universitätsmedizin Greifswald, 17489 Greifswald, Germany; Marco.Franze@med.uni-greifswald.de; 3 IQTIG—Institut für Qualitätssicherung und Transparenz im Gesundheitswesen, 10787 Berlin, Germany

**Keywords:** stress ulcer, acid suppressive therapy, perioperative care, stress ulcer prophylaxis

## Abstract

Background: Despite the growing concern over its potentially severe side effects and considerable economic burden, stress ulcer prophylaxis (SUP) is still frequently prescribed to patients in medical non-intensive care units. Recent data indicate that the situation is similar in surgical departments. Currently, data on the concepts within and regulation of routine SUP practice in surgical departments are sparse. The present study was designed to examine the current practice of SUP in Mecklenburg West Pomerania, Germany, and to identify possible reasons for the dissociation of medical literature and clinical practice. Methods: A questionnaire-based survey was conducted to elucidate current SUP practices in surgical departments of acute care hospitals in Mecklenburg Western Pomerania, Germany. Results: In most surgical departments (68%), a standard operating procedure (SOP) for SUP had not been developed. In departments with an existing SOP, 47.6% of responding medical staff members (MSM) with prescribing authority did not know of its existence. Of the MSMs aware of the existence of an SUP-SOP, only 42.9% indicated that they were familiar with its content. Critical re-evaluation of SUP indications upon transfer from the intensive care unit (ICU) to the general hospital ward (GHW) and before hospital discharge was performed frequently or systematically by only about half of the responding MSMs. Discussion: In the face of continued massive over-prescription of SUP in the perioperative routine, the development of easy-to-use local guidelines and their strict implementation in the clinical routine, as well as intensified medial education on this subject, may be effective tools to reduce acid-suppressive medication (ASM) associated side effects and economic burden.

## 1. Introduction

More than 20 years ago, Gulotta et al. reported the widespread use of acid-suppressive medication (ASM) without adequate indication on hospital wards outside intensive care settings [1]. In the following two decades, multiple publications reported frequencies of inadequate use of ASM in hospitalized internal medicine patients varying from 36.9–100% [2,3,4,5,6,7,8,9]. Inappropriate ASM use has been seen in 67.0–72.6% of hospitalized non-ICU surgical ward patients [10,11]. The protection of the integrity of the gastric mucosa is one of the most common reasons found for non-indicated administration of ASM in patients on GHWs, especially in surgical patients [2,10,11]. The advent of fiberoptic endoscopy led to detection of gastric mucosal lesions in up to 100% of patients in this population, with clinically apparent bleeding in up to 22% of patients [12]. Whilst mortality from gastroduodenal ulceration in the intensive care setting was as high as 58% in this decade, the progress in treatment algorithms and techniques resulted in a compelling reduction in the incidence of clinically important GI bleeding in the ICU to 2.7% [13,14]. Thus, whilst pharmacological SUP was a routine procedure at the end of the 20th century, current guidelines for SUP in the critical care setting recommend against SUP in intensive care patients without risk factors as well as against stress ulcer prophylaxis in GHW patients [15,16,17,18]. Since ASM has long been considered safe and without significant side effects, critics of its widespread use were initially concerned by the cost produced by this practice [7,19]. However, over the last two decades, evidence has accumulated for potentially severe side effects of ASM, including bacterial gastroenteritis [20], acute interstitial nephritis [21], vitamin B deficiency [22], increased risk for COVID-19 [23], community and hospital-acquired pneumonia, dementia, osteoporosis and electrolyte disturbances, to name a few (for review, see Malfertheiner et al. [24]). Both the lack of guidelines recommending routine SUP in non-critically ill patients and the growing list of known side effects of ASM put pressure on hospital practitioners to strictly limit SUP to patients with proven risk factors. However, in a recent study with 1132 surgical patients in a university hospital in the north of Germany, the authors found inappropriate ASM administration in 85.7–99.6% of patients put on de novo SUP during their hospitalization on a surgical non-intensive care ward [25]. These findings clearly show that inappropriate SUP is still a critical issue in current surgical practice in Germany.

Based on these findings, a questionnaire-based survey was designed to examine the current practice of SUP in the federal state of Mecklenburg West Pomerania in order to identify possible reasons for the dissociation of medical literature and clinical practice.

## 2. Materials and Methods

A written questionnaire-based survey on SUP practice in surgical departments in Mecklenburg West Pomerania was conducted between June 2017 and October 2017. The clinical ethics committee of the University Medicine Greifswald approved the study design. The identification of surgical departments in Mecklenburg West Pomerania was based on data contained in the hospital plan of the federal state government of Mecklenburg West Pomerania (Krankenhausplan 2012 des Landes Mecklenburg-Vorpommern, Stand Juni 2016). Departments of general surgery, visceral surgery, vascular and cardiac surgery, orthopedic and trauma surgery, and departments encompassing combinations of these specialties were included in the study. Information on the structure and size of each surgical department was retrieved from the document mentioned above.

Separate questionnaires for the head of department (HoD) and the MSMs were developed in cooperation with the Institute of Community Medicine of the University Medicine Greifswald. The questionnaire for the HoD contained questions concerning the department staffing, the existence of an SOP for SUP, the current practice of SUP in intensive and non-intensive care wards, and medications mainly used for SUP (Supplementary data 1). The questionnaire for MSM contained questions concerning their main professional activity, professional experience, content of and compliance with the SOP for SUP (when present), the personal practice of SUP in clinical routine, and questions on the epidemiology of stress ulcer development and associated risk of clinically relevant gastrointestinal bleeding (Supplementary data 2).

Data was collected from returned questionnaires using a computer-based Access form. Categorical variables were described using frequency. For the comparison of categorical variables, Chi square tests were applied, for small data, the Fischer’s exact test was used. Statistical analysis was conducted using SPSS software version 25.0 (IBM Inc., Armonk, NY, USA).

## 3. Results

### 3.1. Characterization of Responding Heads of Department and Staff Members

Based on the hospital plan of the federal state government of Mecklenburg West Pomerania, 29 acute-care hospitals comprising in total 50 surgical departments were identified, including departments of general surgery, general and visceral surgery, thoracic surgery, orthopedic and trauma surgery, as well as cardiac and vascular surgery. Among the HoDs contacted, 25 responded to the survey (50%). The repartition of the corresponding departments concerning surgical specialty and hospital size is shown in Table 1. A total of 137 MSMs from 30 of the 50 surgical departments contacted returned the questionnaire (60%). The essential characteristics concerning their principal clinical activity, hospital size, and professional experience are summarized in Table 2.

### 3.2. Existence of Standard Operating Procedures Concerning SUP in Surgical Departments

**Figure 1:**
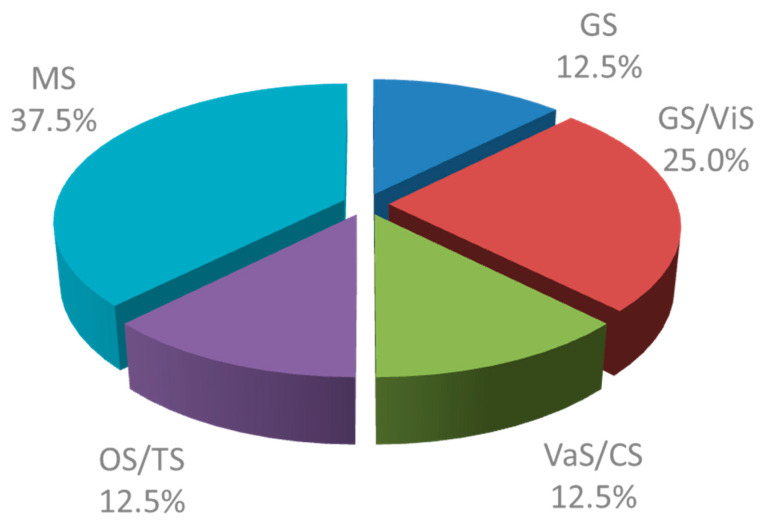
Presence of SOPs for SUP in surgical subspecialties in Mecklenburg West Pomerania, Germany GS: General surgery; GS/ViS: General andVisceral Surgery; VaS/CS: Vascular and cardiac surgery; OS/TS: Orthopedic and trauma surgery; MS: mixed surgical departments.

Among the 25 responding HoDs, only eight affirmed the existence of an SOP for SUP in their department (32.0%). While in none of the small hospitals (<100 beds), an SOP for SUP existed in 37.5% of surgical departments in medium-sized hospitals (100–500 beds) and 33.3% of surgical departments in hospitals with more than 500 beds. However, the departments with SOP in hospitals with more than 500 beds were localized within the same university hospital. The distribution of the SOPs across the surgical specialties is shown in Figure 1. The age of the SOP was unknown in 28.6%, >5 years in 7.1 %, and ≤5 years in 16.7% of cases. The question of SOP age was unanswered by 47.9% of participants. When asked for the prescribing habits for SUP in the intensive care setting, 58.8% of HoDs in departments without SOP and 87.5% of HoDs in departments with SOP answered that intensive care patients were routinely given a SUP. In addition, 23.5% of HoDs of departments without SOP, and 12.5% of HoDs of departments with SOP stated that intensive care patients received a risk-adapted SUP. Outside the intensive care setting, 5.9% of HoDs of departments without SOP and 25% of HoDs of departments with SOP prescribed SUP routinely during hospitalization in the GHW, while patients received a risk-adapted therapy in 47.1% and 50% of departments without and with SOP, respectively (Table 3).

Of the 137 MSMs who returned the questionnaire, 40 surgeons confirmed the existence of an SOP for SUP in their surgical department (29.2%). A total of 47.6% of MSMs working in departments with an existing SOP were not aware of its existence. Of the MSMs in departments with an SOP, 18 (42.9%) confirmed that they had read and were familiar. MSMs working in departments with existing SOPs were asked whether, according to the SOP, the SUP indication was reassessed when patients were transferred from the ICU to the GHW and whether SUP was systematically discontinued when patients were discharged from hospital. In response to the first question, only 28.6% of respondents confirmed that regular reassessment of the indications for SUP continuation after transfer from the ICU to the GHW was included in the SOP. For the second question, only 19% of respondents indicated that SUP discontinuation upon discharge from the hospital was stated in the SOP. However, 47.6% did not answer either question. In response to the first and second question, 7.1% and 21.4% chose the option “others”, respectively (Table 4).

### 3.3. Current Practice of SUP in Surgical Departments in Mecklenburg West Pomerania

**Figure 2:**
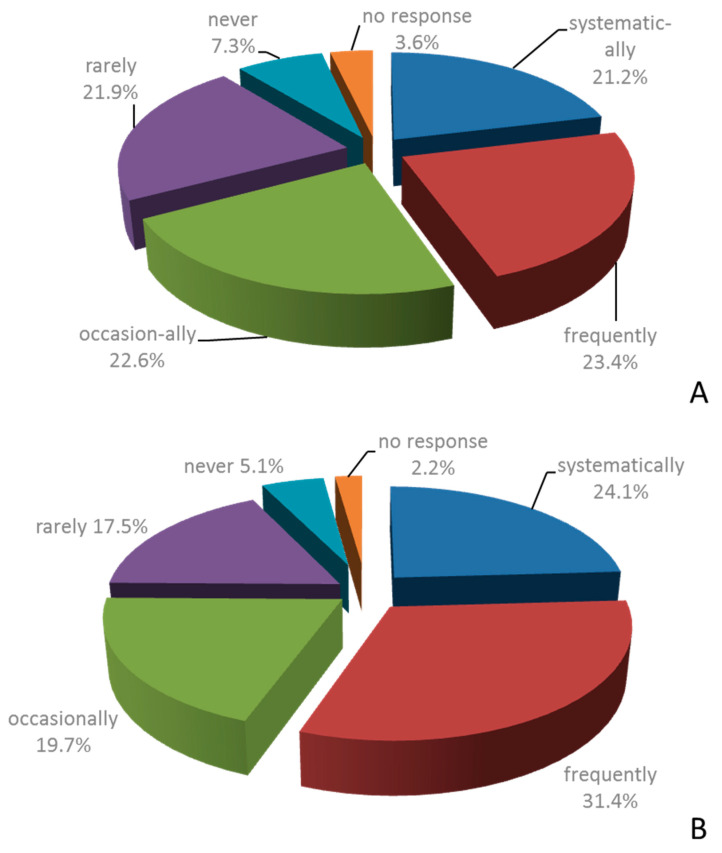
Routine practice of SUP according to MSMs. MSM staff members were asked whether, in their personal daily practice, SUP indication was systematically reassessed on the occasion of the transfer from the ICU/ICM to the GHWs (**A**) and before the discharge of the patient from the hospital (**B**). (*n* = 137).

MSMs of surgical departments in Mecklenburg West Pomerania were asked about SUP in their daily routine. When asked whether the daily routine included a revision of the indication for SUP upon transfer of patients from the ICU/intermediate care station (IMC) to the GHW, only 44.6% of interviewees answered with “systematically” or “frequently”. In this setting, 22.6% and 21.9% responded that the indication was critically reassessed “occasionally” and “rarely”, respectively. Yet, 7.3% of MSMs answered that reassessment of the indication for SUP is “never” conducted in their daily routine (Figure 2). The routine assessment was most frequently done by MSMs whose main activity was in the intermediate care setting and visceral surgery, and most infrequently by MSM whose main activity was in vascular surgery and trauma/orthopedic surgery (Table 5).

When asked whether indications for continued SUP are routinely re-assessed when the patient is discharged from the hospital, 55.5% of MSMs answered “systematically” or “frequently” and 37.2% of interviewees answered with “occasionally” or “rarely”. Only 5.1% indicated that the indication for SUP was never reassessed before discharge (Figure 2). Routine assessment of the indication for continued SUP upon discharge was most frequently done by MSM whose main activity was in the intermediate care setting and visceral surgery, and most infrequently by those whose main activity was in vascular surgery (Table 5).

The most frequently used drug classes for SUP in GHW patients were proton pump inhibitors (93.4%), followed by H2 receptor antagonists (5.8%). 

### 3.4. Individual Knowledge in Stress Ulcer Disease and Prophylaxis

**Figure 3:**
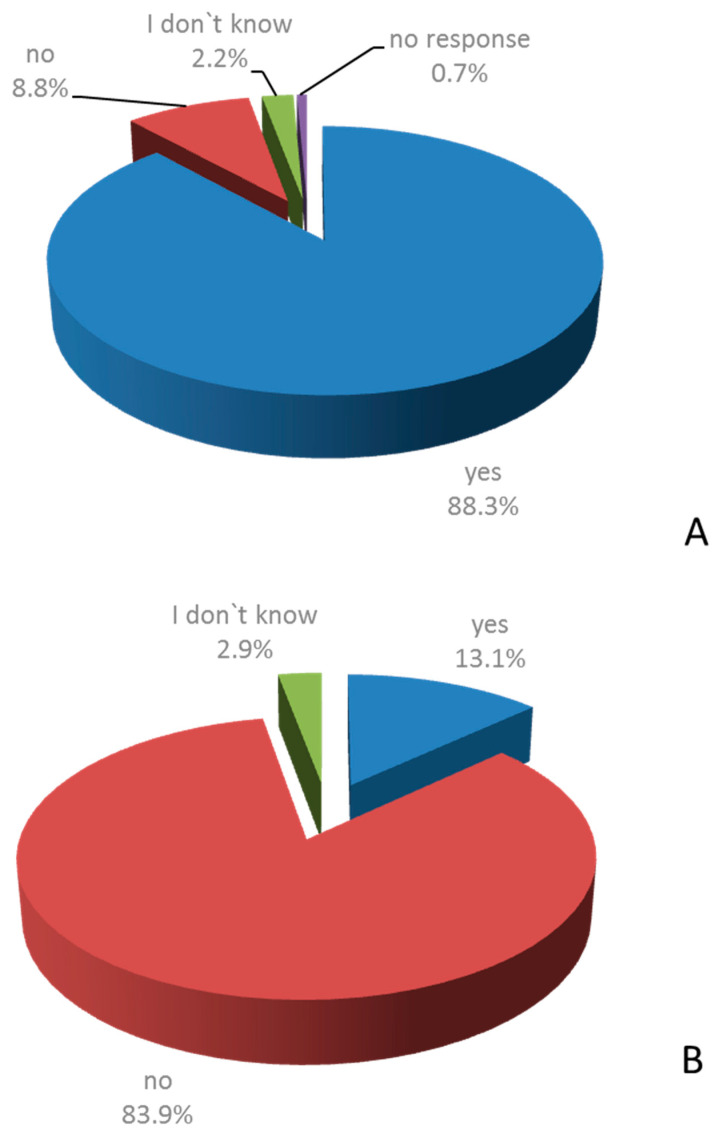
Agreement with statements on SUP in the clinical routine. Staff members were asked whether they agree or disagree with the following statements: “My decision on the prescription of SUP is based on my evaluation of the individual risk of the patients for stress ulcer bleeding” (**A**) and “Due to the high incidence of preventable gastrointestinal bleeding from stress ulcers I prescribe SUP to every hospitalized patient.” (**B**) (*n*= 137).

MSMs were asked to agree or disagree with the following two statements: “My decision on the prescription of SUP is based on my evaluation of the individual risk of the patients for stress ulcer bleeding.” and “Due to the high incidence of preventable gastrointestinal bleeding from stress ulcers I prescribe SUP to every hospitalized patient.” A total of 88.3% of MSMs agreed with the first statement, while 8.8% of MSMs disagreed. A total of 13.1% of MSMs agreed with the second statement; 83.9% disagreed (Figure 3). 

**Figure 4:**
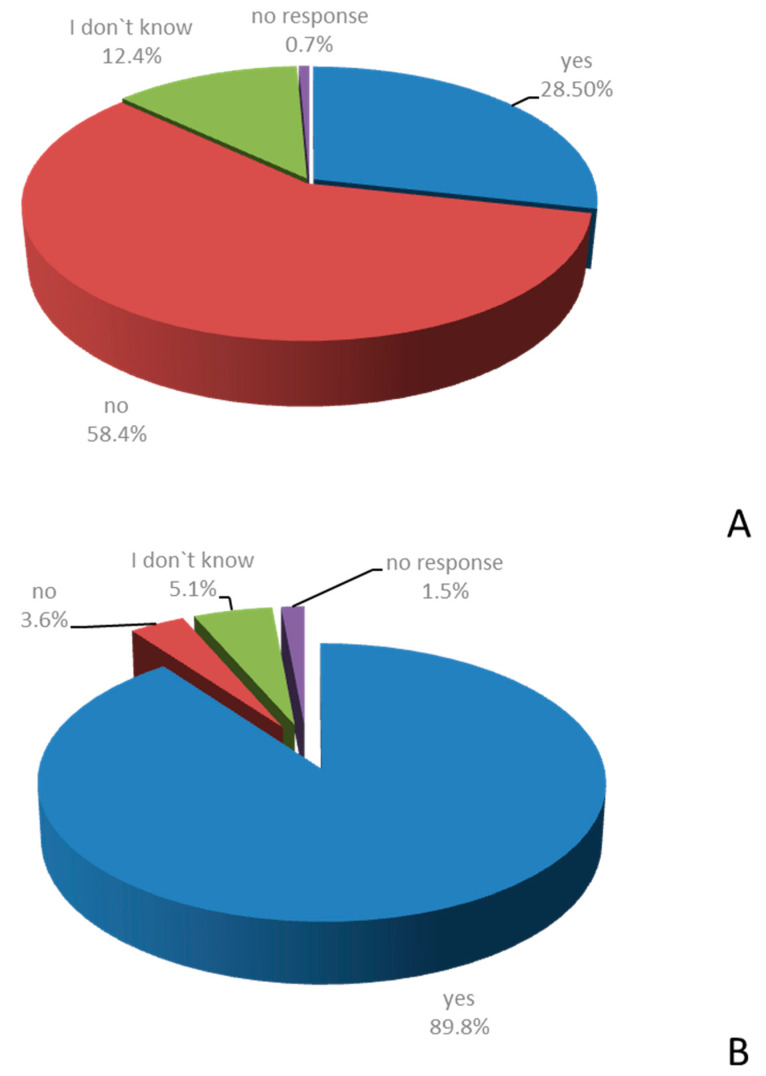
Agreement with statements on the personal experience with SUP in the clinical routine. MSMs were asked whether they agree or disagree with the following statements: “I feel unconfident when asked to assess the risk of bleeding from stress ulcers in individual patients.” (**A**) and “Do you feel aware of the benefits and risks of SUP?” (**B**) (*n* = 137).

Although 88.3% of MSMs agreed to prescribe SUP based on their personal appraisal of the individual patient’s risk for developing bleeding from stress ulcers, as many as 28.5% responded “yes” to the following statement: “I feel unconfident when asked to assess the risk of bleeding from stress ulcers in individual patients”. To this same statement, 58.4% responded “no” (Figure 4A).

To the question: “Do you feel aware of the benefits and risks of SUP?” 89.8% of participants answered: “yes,” while 3.6% responded with “no” (Figure 4B). 

Most recent publications report a 2.7% incidence of stress ulcer-induced bleeding in intensive care patients [26]. When asked to estimate the incidence of stress ulcer-induced bleeding in intensive-care patients, only 20.4% of the answers fell within an accepted error range of 50% around this value. Totals of 64.9% and 11.7% of participants overestimated and underestimated the incidence of stress ulcer-induced bleeding, respectively (Table 6).

When asked whether official guidelines exist for SUP in intensive care patients, only 18.2% of medical general ward patients and surgical general ward patients correctly answered that there is only a guideline for SUP in intensive care patients. By far the highest percentage of respondents (46.0%) admitted to not knowing whether a guideline exists for any of these patient groups.

## 4. Discussion

SUP remains part of clinical routine in a substantial number of non-ICU surgical wards. Bez et al. reported that up to 54% of patients admitted to a surgical ward without prior ASM were started on SUP in 2010 [10]. In a mixed medical-surgical patient population, Parente et al. reported the start of ASM in 37% of patients upon admission to the hospital [11]. Our research group recently assessed the ASM prescription routine for SUP on non-ICU surgical wards in a German university hospital: SUP was initiated in 40.3% of patients admitted to the normal surgical ward, and for 85.7–99.6% of those patients, no adequate indication for SUP could be identified retrospectively [25]. Thus, although the first report on the non-indicated over-prescription of ASM for SUP was published more than 20 years ago [1], and although a plethora of literature documents this phenomenon in medical and intensive care patients, current surgical perioperative practice seems as yet unaffected by this scientific evidence. The findings reported in this work provide some information on the origins of this situation.

In the absence of a national guideline on SUP in surgical non-ICU patients, local SOPs that define the principles of perioperative SUP may be an efficient tool to prevent inappropriate use of ASM for SUP in surgical patients. However, they exist only in the minority of hospitals in Mecklenburg West Pomerania. Notably, in small hospitals with a size <100 beds, SOPs for perioperative SUP were absent. However, even in those departments where the HoD confirmed the existence of an SOP, only 52.4% of SMs were aware of its existence, and only 42.9% confirmed having read the document. Thus, even in departments where care was taken to create an SOP, compliance appears to be insufficient. Based on the statements of the MSMs, systematic reassessment of the indications for continued SUP upon transmission from the ICU/IMC to the GHW was part of the SOPs contained in less than 50% of cases. Accordingly, when asked for their personal routine, only 44.6% of responding MSM answered that they “routinely” or “frequently” assessed the indications for continued SUP in this setting. This practice contrasts with recommendations for the discontinuation of SUP after transferal to the GHW [17]. In clinical practice, 80% of patients were transferred from the ICU to the GHW with continued ASM, 60% of which were non-indicated [27]. Other authors found that up to 86.7% patients discharged from ICUs were unnecessarily exposed to ASM [28]. A lack of regular reassessment of SUP indication may be one critical point responsible for SUP overuse since patients sojourning on the IMC during hospital stays are more likely to be started on non-indicated SUP than patients on GHWs [25]. 

Routine discontinuation of SUP upon discharge from hospital was mentioned in less than 20% of SUP SOPs. Fortunately, more than 55% of MSMs indicated that reassessment of indications for continued SUP after hospital discharge was part of their personal routine. Repetitious reassessment of the indications for SUP in hospitalized patients is a prerequisite for the reduction of SUP overuse, especially against a background of increasing concerns over the side effects of ASM [20,21,22,24,29,30,31]. Our findings indicate a lack of implementation of local guidelines for SUP, including a lack of definition of critical checkpoints for the reassessment of SUP necessity during the hospital stay. Development and efficient implementation of guidelines and measures to increase compliance could improve perioperative ASM prescription practice. 

The majority of MSMs indicated that, in their personal daily practice, SUP prescription is based on their personal appraisal of the individual patient risk for developing stress ulcers. Only 58.4% of MSMs disagreed with the following statement: “I feel unconfident when asked to assess the risk of bleeding from stress ulcers in individual patients”. This finding indicates the presence of significant uncertainty surrounding the correct indication for SUP. Moreover, in our survey, the risk for stress ulcer-related bleeding was massively overestimated, confirming previous findings indicating that although the relative risk of developing stress ulcer-related bleeding can be correctly assessed by the treating physician, the absolute risk is generally overestimated [25]. Compared with previous surveys, which showed a critical overestimation of the incidence of stress ulcer-induced bleeding in less than 50% of participants, the current investigation revealed a far more pronounced overestimation rate of 64.9%. These findings indicate that more intensive continued medical education on stress-induced ulcer disease, its clinical relevance, clinical risk factors, and indications for prophylactic medical treatment is required. Interventions involving intensified medical education have been shown to effectively reduce both the overall use and the non-indicated use of ASM in medical non–intensive care settings [32].

The correct indication for ASM in hospitalized non-ICU patients is complex. Evidence for and against the prophylactic administration of ASM is based on different treatment recommendations for specific disease entities or on extrapolations of recommendations for intensive-care patients of different medical societies, e.g., Surviving Sepsis Campaign, the German Society for Digestive and Metabolic Diseases (DGSV), the German Society of Cardiology, Danish Society of Intensive Care Medicine, and the Danish Society of Anesthesiology and Intensive Care Medicine [16,18,33,34] as well as on individual scientific publications [6,35]. To date, there is no specific SOP for SUP in non-ICU patients. This emphasizes the importance of developing a national guideline or at least local practice guidelines to systemize available evidence and translate it into uniform and coherent clinical practice. Additionally, continued medical education on this issue is required to implement this guideline into clinical routine.

Local implementation of pharmacologist-developed guidelines has been shown to reduce SUP administration in non-surgical, non-ICU wards in up to 45% of cases [36]. Similarly, in the critical care setting, Coursol et al. reported a reduction in inappropriate SUP use after the implementation of a local guideline [37]. After the introduction of a practice guideline in the ICU setting, Moustafa et al. observed a reduction in overall-SUP use, of the percentage of inappropriate SUP use as well as a reduction in the economic burden caused by non-indicated SUP [38].

The participation of pharmacists in the decision process about the initiation of SUP, both in an intensive care setting and a non-intensive care setting either by chart review or by participating in interdisciplinary ward rounds, had a significant positive impact on both overall and inappropriate use of SUP as well as on ASM-associated inpatient costs [32,36,39,40,41,42]. However, this strategy will not be an option in small country hospitals with limited personnel and financial resources. In this context, intensified clinical educational programs may partly compensate for the absence of clinical pharmacists [32].

Several limitations of the present study need to be addressed when interpreting the results. First, the return rate of the questionnaires for HoDs and MSMs covered only 50% and 60% of surgical departments in Mecklenburg West Pomerania, respectively. When interpreting the results, it should be considered that this response rate may have induced a selection bias. Second, direct questions as used in our questionnaire may induce answers which are judged socially positive by the respondent. Thus, possibly, the measurement might have been affected by a social desirability bias, resulting in a too positive picture of clinical reality of SUP in Mecklenburg Western Pomerania.

## 5. Conclusions

The results reported herein reveal a lack of SUP standardization, e.g., in SOPs, and, if SOPs exist, a lack of their implementation in clinical routine. Since the indication for ASM administration perioperatively is increasingly complex, developing a national SUP guideline or, alternatively, at least standardized local SUP protocols appears necessary. Furthermore, since our results show an insufficient implementation of already existing SUP SOPs in clinical routine, the provision of continued medical education on this subject is a second vital prerequisite to improve the appropriateness of perioperative SUP management in surgical non-ICU patients.

## Figures and Tables

**Table 1:** Characteristics of surgical departments with HoD participating in the survey (*n* = 25).

Type of Department	Number (%)
General Surgery	1 (4)
General and Visceral Surgery	5 (20)
Vascular and Cardiac Surgery	1 (4)
Orthopaedic and Trauma Surgery	8 (32)
Mixed Surgical Departments	10 (40)
Size of Hospital (beds)	Number (%)
<100	3 (12)
100–500	16 (64)
>500	6 (24)

**Table 2:** Characteristics of MSM participating in the survey concerning principal professional activity, professional experience, and size of hospital (*n* = 137).

	HoD Responding *n* = 120 (%)	HoD Not Responding *n* = 17 (%)	
Principal professional activity			
General Surgery	42 (35)	13 (76.5)	*p* = 0.05
Visceral Surgery	16 (13.3)	1 (5.9)	
Vascular and Cardiac Surgery	12 (10)	1 (5.9)	
Orthopaedics and Trauma	41 (34.2)	2 (11.7)	
Intermediate Care/Intensive Care	5 (4.2)	0 (0)	
Others	4 (3.3)	0 (0)	
No answers	0 (0)	0 (0)	
Size of Hospital (beds)			
<100	9 (7.5)	7 (41.2)	*p* = 0.00
100–500	64 (53.3)	2 (11.7)	
>500	47 (39.2)	8 (47.1)	
Professional experience			
Resident < 2 years (Assistenzarzt)	15 (12.5)	3 (17.6)	*p* = 0.55
Resident > 2 years (Assistenzarzt)	37 (30.8)	4 (23.5)	
Specialist	29 (24.2)	2 (11.8)	
Consultant	39 (32.5)	8 (47.1)	

**Table 3:** Routine SUP in the intensive care unit and the GHW of participating surgical departments according to the HoD response to the question: “Do of patients in the ICU/GHW receive SUP routinely?” (*n* = 25).

	Departments with SOP for SUP (*n* = 8)	Departments without SOP for SUP (*n* = 17)	
Intensive care unit			
no answer	0	1 (5.9)	*p* = 0.49
yes	7 (87.5)	10 (58.8)	
no	0	2 (11.8)	
risk adapted	1 (12.5)	4 (23.5)	
General hospital ward			
no answer	0	1 (5.9)	*p* = 0.46
yes	2 (25)	1 (5.9)	
no	2 (25)	7 (41.2)	
risk adapted	4 (50)	8 (47.1)	

**Table 4:** Content of the SUP-SOP according to the MSM answers. MSMs confirming the existence of a SUP in their departments were asked whether routine reassessment of the indication for SUP transfer from the ICU/ICM to the GHWs and whether SUP discontinuation after discharge was specified in the SOP. Percentages were calculated in relation to the number of MSMs confirming the existence of an SOP in their department (*n* = 42).

	Reassessment of Indications for SUP upon Transfer from the ICU to the GHW According to SOP *n* = 42	Discontinuation of SUP after Hospital Discharge According to SOP *n* = 42
yes	12 (28.6)	8 (19.1)
no	7 (16.7)	5 (11.9)
no answer	20 (47.6)	20 (47.6)
other	3 (7.1)	9 (21.4)

**Table 5:** Routine practice of SUP according to MSMs. MSMs were asked whether, in their personal daily practice, SUP indication was systematically reassessed on transfer from the ICU/ICM to the GHWs and before the discharge of the patient from the hospital. Results are classified according to the leading professional activity of the respondents (*n* = 137).

	General Surgery *n* = 55	Visceral Surgery *n* = 17	Vascular Surgery *n* = 13	ICU/IMC *n* = 5	Trauma/ Orthopaedics *n* = 43	Others *n* = 4	
Reassessment of SUP indications upon transfer from ICU to normal ward							
systematically	8 (14.5)	6 (35.3)	1 (7.7)	1 (20)	13 (30.2)	0	
frequently	17 (30.9)	5 (29.4)	4 (30.8)	3 (60)	3 (7.0)	0	
occasionally	15 (27.3)	4 (23.5)	4 (30.8)	0	8 (18.6)	0	*p* = 0.00
rarely	12 (21.8)	1 (5.9)	3 (23.1)	0	13 (30.2)	1 (25.0)	
never	2 (3.6)	0	0	0	5 (11.6)	3 (75.0)	
no response	1 (1.8)	1 (5.9)	1 (7.7)	1 (20)	1 (2.3)	0	
Reassessment of SUP indications before hospital discharge							
systematically	11 (20.0)	8 (47.1)	2 (15.4)	3 (60.0)	9 (20.9)	0	
frequently	22 (40.0)	5 (29.4)	2 (15.4)	2 (40.0)	12 (27.9)	0	
occasionally	13 (23.6)	4 (23.5)	5 (38.5)	0	5 (11.6)	0	*p* = 0.01
rarely	7 (12.7)	0	4 (30.8)	0	10 (23.3)	3 (75.0)	
never	1 (1.8)	0	0	0	5 (11.6)	1 (25.0)	
no response	1 (1.8)	0	0	0	2 (4.7)	0	

**Table 6:** Perceived incidence of stress-ulcer induced GI-bleeding. Participating MSMs were asked to estimate the incidence of stress ulcer-induced GI bleeding in intensive care patients. Answers within an error range of ±50% of the incidence found in the literature (2.7%) 26 were considered correct.

	% of Participating MSMs (*n* = 137)
Underestimation of more than 50%	16 (11.7)
Correct estimation	28 (20.4)
Overestimation of more than 50%	32 (23.4)
Overestimation of more than 100%	3 (2.1)
Overestimation of more than 200%	54 (39.4)
No answer	4 (2.9)

## Data Availability

Primary data can be obtained by the corresponding author upon request.

## Supplementary Materials

The following are available online at https://www.mdpi.com/article/10.3390/healthcare9111490/s1, Supplementary data 1: Questionnaires addressed to the heads of surgical departments in acute care hospitals in Mecklenburg West Pomerania (English translation); Supplementary data 2: Questionnaires addressed to the MSMs of surgical departments in acute care hospitals in Mecklenburg West Pomerania (English translation).
