# Supplementary material for: Current Practice of Stress Ulcer Prophylaxis in Surgical Departments in Mecklenburg Western Pomerania, Germany
Source: Healthcare (Basel). 2021 Nov 2;9(11):1490. doi: 10.3390/healthcare9111490 (PMC8625761; doi:10.3390/healthcare9111490)
Supplement: Supplementary file 1 [file healthcare-09-01490-s001.zip › Supplementary data 1_ Rauch et al rev 2 2021029.pdf]

Questionnaire for the heads of surgical departments in Mecklenburg West Pomerania

Questionnaire A Stress ulcer prophylaxis

1) In our department, is there a written standard operating procedure (SOP) describing the indications and the protocol for stress ulcer prophylaxis (SUP)
   - ☐ Yes
   - ☐ no
   - ☐ others (Please describe the details below)

   Commentary: ____

   ____

2) Do patients in the ICU/GHW receive SUP routinely??

	yes	no	risk-adapted (Please describe the details below)	others (Please describe the details below)
Normal ward				
Intensive care ward				

Commentary: ____

____

Which drug class is predominantly used for SUP in your department (Please chose one option)?

- ☐ H2-blockers
- ☐ Protone pump inhibitors
- ☐ others (Please describe the details below)

Commentary:____

____
