# Supplementary material for: Current Practice of Stress Ulcer Prophylaxis in Surgical Departments in Mecklenburg Western Pomerania, Germany
Source: Healthcare (Basel). 2021 Nov 2;9(11):1490. doi: 10.3390/healthcare9111490 (PMC8625761; doi:10.3390/healthcare9111490)
Supplement: Supplementary file 1 [file healthcare-09-01490-s001.zip › Supplementary data 2_ Rauch et al 20210925.pdf]

Questionnaire for the staff of surgical departments in Mecklenburg West Pomerania

Questionnaire B Stress ulcer prophylaxis

1. In which hospital you are currently employed?

   ....

2. What is the level of your professional experience?

   ☐ Senior physician
   ☐ Specialist
   ☐ Senior house officer during the last four years of surgical training
   ☐ Senior house officer during the first two years of surgical training

3. What is the surgical specialty in which you are predominantly working?

   ☐ General Surgery
   ☐ Visceral Surgery
   ☐ Vascular Surgery
   ☐ Thoracic Surgery
   ☐ Unfallchirurgie/Orthopädie
   ☐ Trauma/Orthopedics
   ☐ Others (Please specify):

4. Is there a standard operating procedure on stress ulcer prophylaxis in your hospital?

☐ yes

☐ no

☐ others (Please describe the details below):

Commentary ______________________________

____

If you have answered "Yes "to question 4, please answer questions 5-8, too.	If you have answered "No "to question 4, please continue with question 9.

5. When was the SOP on stress ulcer prophylaxis implemented?

☐ month/year: ...../......

☐ unkown

6. Are you familiar with the content of the SUP, i.e., have you entirely read the SUP?

☐ yes

☐ no

☐ others (Please describe the details below):

commentary: ______________________________

____

7. According to the SOP, is there systematic discontinuation of SUP when patients are discharged from the hospital?

☐ yes

☐ no

☐ others (Please describe the details below):

Commentary: ____

____

8. According to the SOP, is there systematic re-evaluation of the indications for SUP when patients are transferred from the ICU to the normal ward?

- ☐ yes
- ☐ no
- ☐ others (Please describe the details below):

Commentary ____

____

9. Personally, do you actively re-evaluate the indications for SUP when transferring a patient from an intensive care unit to the normal ward, and do you discontinue the medication when SUP is no longer indicated?

- ☐ never
- ☐ rarely
- ☐ occasionally
- ☐ frequently
- ☐ systematically

Commentary: ____

____

10. Personally, do you re-evaluate the indication for acid-suppressive medication when discharging patients at home, and do you discontinue acid-suppressive medication if the indication is no longer present?

☐ never
☐ rarely
☐ occasionally
☐ frequently
☐ systematically

Commentary ____

____

11. Which drug class is predominantly used for SUP in your department (Please chose one option)?

☐ H2-blockers
☐ Protone pump inhibitors
☐ others (Please describe the details below)

Commentary ____

____

12. What is your estimation for the incidence of episodes of clinically relevant GI-Bleedings in intensive care patients?

.... (%)

13. What is your estimation of the percentage of episodes of upper GI-Bleeding that can not be prevented by stress ulcer prophylaxis in intensive care patients?

.... (%)

13. There are official recommendations for stress ulcer prophylaxis in the following patient groups (multiple choices are possible.)
    a) intensive care patients
    b) internal medicine patients in the normal ward setting
    c) surgical patients in the normal ward setting
    d) for non of the three patient groups above
    e) do not know

14. Please indicate whether or not you agree with the following statements:

	yes	no	Do not know
My decision on the prescription of SUP is based on my evaluation of the patients' individual risk for stress ulcer bleeding.			
Do you feel aware of the benefits and risks of SUP?			
Due to the high incidence of preventable gastrointestinal bleeding from stress ulcers, I prescribe SUP to every hospitalized patient			
I feel unconfident when asked to assess the risk of bleeding from stress ulcers in individual patients.			
